# Design and Fabrication of Air-Coupled CMUT for Non-Contact Temperature Measurement Applications

**DOI:** 10.3390/mi16091008

**Published:** 2025-08-31

**Authors:** Xiaobo Rui, Yongshuai Ma, Chenghao He, Chi Zhang, Zhuochen Wang, Hui Zhang

**Affiliations:** 1 State Key Laboratory of Precision Measuring Technology and Instruments, Tianjin University, Tianjin 300072, China; ruixiaobo@tju.edu.cn (X.R.); mys2020@tju.edu.cn (Y.M.); hch302660444@163.com (C.H.); czhang2023@tju.edu.cn (C.Z.); 2 College of Mechanical and Electrical Engineering, Beijing University of Chemical Technology, Beijing 100029, China; zc.wang@buct.edu.cn

**Keywords:** capacitive micromachined ultrasonic transducer (CMUT), acoustic temperature measurement, temperature field imaging

## Abstract

Compared with traditional piezoelectric transducers, Capacitive Micromachined Ultrasonic Transducers (CMUTs) have advantages such as better impedance matching with air, smaller size, lighter weight, higher sensitivity, and ease of array formation. Acoustic temperature measurement is a technology that utilizes the relationship between sound velocity and temperature to achieve non-contact temperature detection, with advantages such as fast response and non-invasiveness. CMUT-based acoustic temperature field measurement can achieve temperature detection in situations with narrow spaces, portability, and high measurement accuracy. This paper investigates an air-coupled CMUT device for acoustic temperature measurement, featuring a resonant frequency of 220 kHz, and composed of 16 × 8 cells. The design and fabrication of the CMUT array were completed, and the device characteristics were tested and characterized. A temperature field measurement method using mechanical scanning was proposed. A temperature measurement experimental system based on CMUT devices was constructed, achieving preliminary measurement of acoustic transmission time in both uniform and non-uniform temperature fields. Using a temperature field reconstruction algorithm, the measurement and imaging of the temperature field above an electric heating wire were accomplished and compared with the thermocouple-based temperature measurement experiment. The experimental results verified the feasibility of CMUT devices for non-contact temperature field measurement.

## 1. Introduction

A CMUT (Capacitive Micromachined Ultrasonic Transducer) is a thin-film ultrasonic transducer driven by electrostatic force, with advantages such as low cost in mass production, miniaturization, and high sensitivity [1,2]. As a sensor for air-coupled ultrasonic technology, CMUT uses air as a couplant, providing non-contact, environmentally friendly, and wide applicability [3,4]. It is widely used in structural health monitoring, non-destructive testing [5], distance measurement [6], wind speed measurement [7], temperature measurement [8], and gas concentration measurement [9].

Ultrasonic temperature measurement technology utilizes the relationship between the sound speed and temperature to achieve temperature measurement [10]. Compared with traditional contact-based methods, it offers faster response, non-contact operation, and minimal interference with the measurement area. Temperature field imaging in specific regions can be achieved using reconstruction algorithms. Non-contact ultrasonic temperature measurement methods can be used not only to measure the temperature of specific gas or liquid environments [11,12,13] but also to determine the temperature of biological tissues or solid materials [14,15,16]. Previous researchers have mainly focused on temperature field imaging algorithms, optimization of the signal processing process, and the development of temperature measurement systems. In these studies, traditional piezoelectric transducers were primarily employed, with limited exploration of newer transducers like CMUT or PMUT (Piezoelectric Micromachined Ultrasonic Transducer).

Tat Hean Gan et al. [17] reconstructed the temperature field of a high-temperature flame in a natural gas burner using CMUT and a filtered back-projection algorithm. Peter Holsteint et al. [18] studied acoustic temperature tomography based on audible sound, using algebraic reconstruction techniques to invert sound velocity data and calculate flow field temperature. William M. D. Wright et al. [19] utilized a pair of air-coupled electrostatic ultrasound transducers with divergent sound fields to reconstruct air containing solids, airflow, and temperature fields. Zhichun Shao et al. [20] used PMUT devices to achieve non-contact temperature measurements of different areas above a heating plate. Michael Schwarz et al. [21] studied the Tikhonov regularization reconstruction algorithm and compressive sensing algorithm, further optimizing the accuracy of temperature distribution reconstruction under multiple heat source conditions. Li Zhao et al. [22] proposed a reconstruction algorithm based on inverse quadratic function and singular value decomposition, which can reduce the reconstruction error by 0.49%. Mantalena Sarafianou et al. [23] used PMUT devices to measure ambient temperature and achieved high-precision time delay estimation of the received signal using matched filtering.

Conventional piezoelectric probes suffer from significant energy loss due to impedance mismatch with air, which can be effectively addressed by using air-coupled CMUT devices. CMUT devices also have the advantages of small size, light weight, and ease of integration with backend circuits. The MEMS manufacturing process allows flexible customization of CMUT arrays for specific temperature measurement scenarios to ensure better measurement results. Additionally, CMUT devices easily achieve high-frequency operation, ensuring that CMUT has high detection accuracy in temperature measurement applications. Therefore, CMUT-based acoustic temperature field measurement has significant research value for applications requiring limited space, portability, and high accuracy, such as non-contact biological tissue temperature measurement, micro-reaction container monitoring, and microbial culture environments.

This paper presents a capacitive micromachined ultrasonic transducer for acoustic temperature measurement in an air medium. Based on the principal characteristics of air-coupled CMUT, the structural parameters are designed. The device is fabricated and characterized in terms of geometric and impedance parameters, as well as transmissions and reception performance of the CMUT array. Reasonable excitation methods for the device are also explored. Furthermore, an ultrasonic temperature measurement scheme using the CMUT is investigated, and the feasibility of temperature field imaging is demonstrated experimentally.

## 2. Device Design and Fabrication

### 2.1. Device Parameters

**Figure 1:**
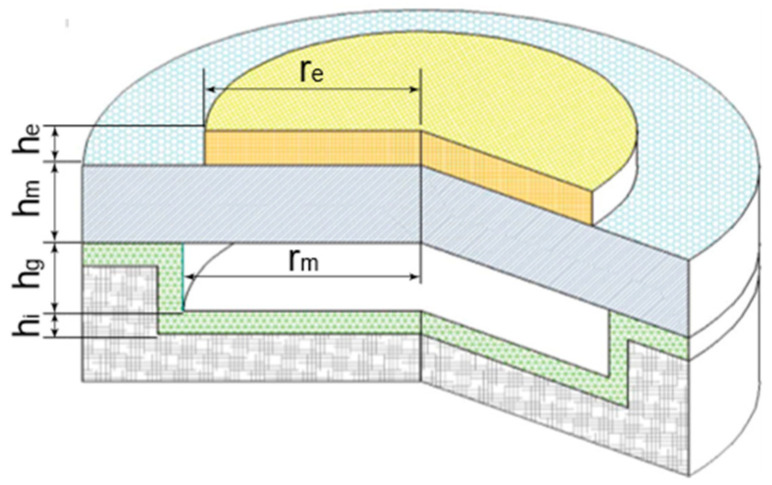
Schematic diagram of the geometric structure of a circular CMUT cell.

A circular CMUT array consisting of 16×8 cells was designed for temperature measurement in this paper; Figure 1 shows the geometric structure of a microelement with a electrode radius *re*, electrode thickness *he*, membrane radius *rm*, membrane thickness *hm*, vacuum chamber depth *hg*, and insulation layer thickness *hi*. The attenuation of the ultrasonic wave is proportional to the propagation distance and the square of the ultrasonic frequency. For balance detection distance and resolution, the operating frequency of 220 kHz was designed. The resonant frequency of CMUT is inversely proportional to the membrane radius and proportional to the membrane thickness. After comprehensive consideration, the CMUT membrane size with a radius of 400 μm and a thickness of 10 μm was finally determined. Similarly, considering that CMUT can be used as a transmitter or a receiver, the cavity depth is designed to be 5 μm. In summary, the CMUT structural parameters are shown in Table 1.

### 2.2. Finite Element Simulation

#### 2.2.1. Pull-in Characteristics Analysis

**Figure 2:**
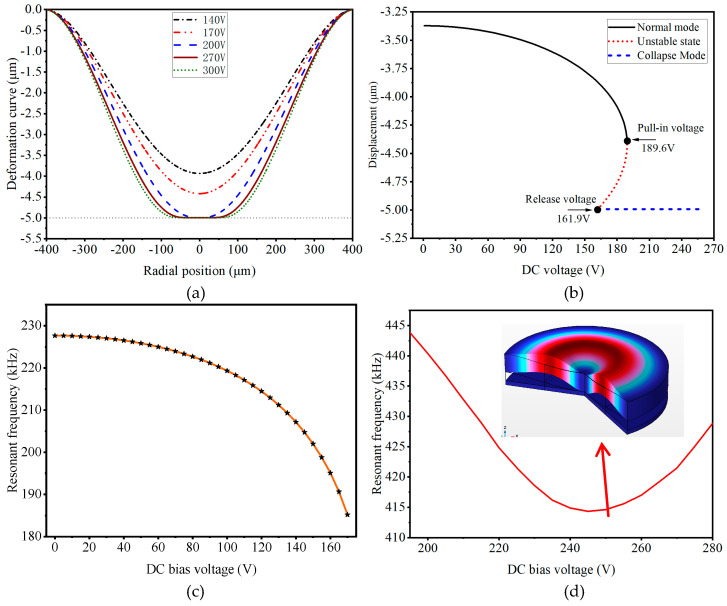
Analysis of CMUT suction state characteristics: (**a**) The relationship between membrane deformation and voltage. (**b**) The relationship between membrane center displacement and voltage. (**c**) The relationship between resonant frequency and DC voltage in the non-pull-in state. (**d**) The relationship between resonant frequency and DC voltage in the pull-in state.

Figure 2a shows the relationship between DC voltage and membrane deformation by COMSOL 6.2 simulation. As the DC voltage increases, the membrane gradually approaches the substrate. When the DC bias continues to increase, the electrostatic force acting on the membrane exceeds its elastic restoring force, causing the membrane to adhere to the bottom electrode. This state is usually called the electrostatic pull-in state. Figure 2b shows the relationship between the displacement of the center of the membrane and the change in voltage. The device’s pull-in voltage is 189.6 V, and the release voltage is 161.9 V.

Figure 2c shows the relationship between the DC voltage and the first-order resonant frequency in the non-pull-in state. As the voltage increases, the resonant frequency of the membrane decreases. Figure 2d illustrates the frequency–voltage relationship in the pull-in state. When the voltage changes from 195 V to 245 V, the resonant frequency gradually decreases. However, when the voltage changes from 245 V to 280 V, the resonant frequency of the membrane begins to increase again, which is caused by the stiffness increase of the membrane under large deformation. The color chart in Figure 2d shows the vibration mode of CMUT under a DC voltage of 250 V. At this point, the center of the membrane will be electrostatically adsorbed onto the substrate, with a resonant frequency of 414.62 kHz, which is almost twice the non-contact resonant frequency.

#### 2.2.2. Response Characteristic Analysis

**Figure 3:**
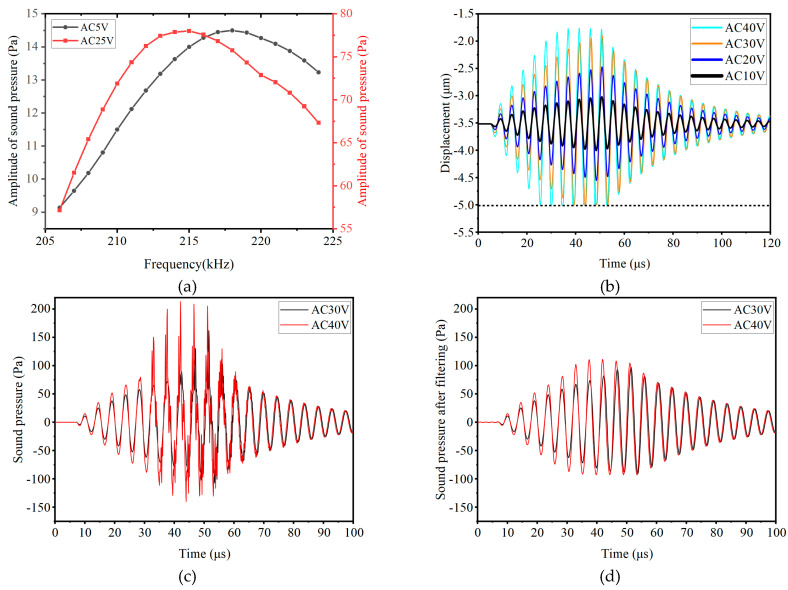
The excitation response characteristics of CMUT: (**a**) Relationship between AC amplitude and resonant frequency. (**b**) Displacement response of the CMUT membrane under different AC. (**c**) Original sound pressure signal. (**d**) Filtered sound pressure signal.

A DC bias of 100 V, along with 10 sinusoidal pulse bursts of 5 V and 25 V, was applied to the CMUT. The sound pressure response at 940 μm in front of the membrane was simulated for frequencies from 206 kHz to 224 kHz. Figure 3a shows that higher AC amplitudes reduce the frequency of maximum sound pressure due to the spring softening effect.

Figure 3b shows the membrane center displacement for different AC amplitudes at 216 kHz. As the excitation signal increases, the vibration range of the membrane center also increases. When the AC amplitude reaches or exceeds 30 V, the membrane displacement reaches -5 μm, where the membrane comes into contact with the bottom electrode.

The emission sound pressure of CMUT was also simulated, as shown in Figure 3c, which depicts the sound pressure signal at 1 mm in front of the membrane. When dynamic contact occurs between the membrane and the substrate, high-frequency noise is generated due to the membrane’s collision with the substrate. After applying a 500 kHz low-pass filter to remove this high-frequency noise, the filtered result is shown in Figure 3d. It can be seen that the emitted sound pressure under an AC of 40 V is greater than that at 30 V, consistent with the trend of the average displacement changing with the excitation signal.

### 2.3. Device Testing

#### 2.3.1. Topography Measurement

**Figure 4:**
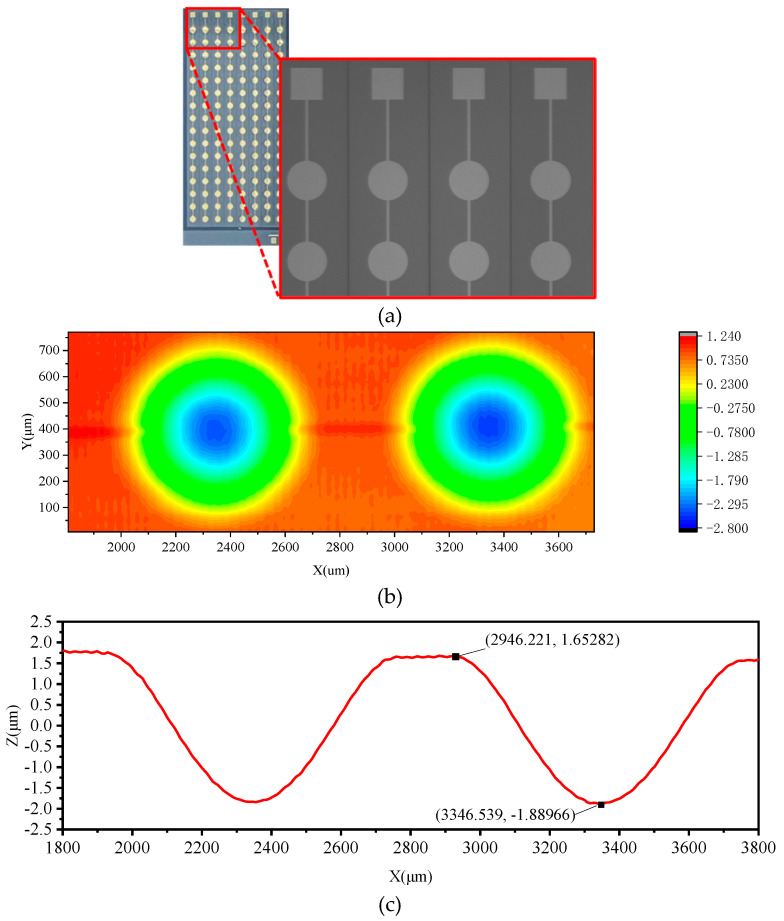
CMUT topography measurement results: (**a**) 8 × 16 CMUT array and microscope image, (**b**) CMUT cell morphology measurement image, and (**c**) deformation of CMUT cell cross-section.

The CMUT was fabricated by the Si-SOI bonding process. First, a cavity was etched into the silicon wafer using Deep Reactive Ion Etching (DRIE). The etched silicon wafer was then oxidized to form an insulating layer. Subsequently, it was bonded to an SOI wafer. After that, the silicon substrate and the buried oxide layer of the SOI wafer were selectively removed by wet etching. The remaining silicon layer was then etched to form the isolation trenches between elements. Finally, the metal was sputtered as the upper electrode. Figure 4a shows the fabricated CMUT and a microscope image. A white light profilometer was used to scan and measure the cell profiles of the CMUT array, as shown in Figure 4b. The deformation of the section line along the center of adjacent cells is shown in Figure 4c, where the displacement of the membrane center relative to the bonding position is 3.54 μm, while the displacement calculated through the simulation model is 3.39 μm. The finite element simulation model can accurately predict the deformation characteristics of the membrane, and the individual cells in the array exhibit good consistency.

#### 2.3.2. Impedance Test

**Figure 5:**
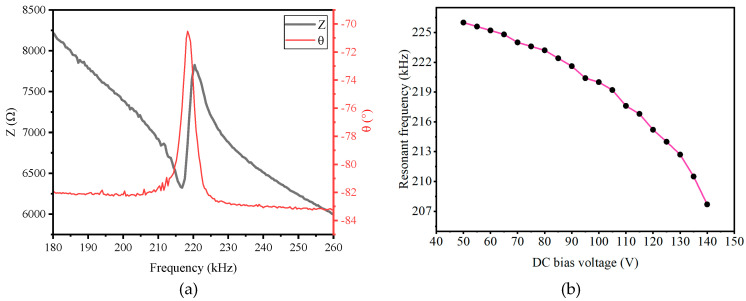
Impedance test results: (**a**) impedance characteristics curve of the CMUT array under 100V DC, and (**b**) relationship between impedance parameters and voltage.

A precision impedance analyzer (Agilent 4294A, Tallahassee, FL, USA) was used to test the impedance of the CMUT elements. Under a DC bias voltage of 100 V, the impedance of a single element is shown in Figure 5a, where the resonant frequency corresponds to the peak of the phase resonance. By changing the DC voltage and measuring the resonant frequency, the curve in Figure 5b was obtained, showing that the phase resonance peak shifts toward lower frequencies as the DC voltage increases, which is consistent with the simulation results.

#### 2.3.3. Pull-in Voltage Test

**Figure 6:**
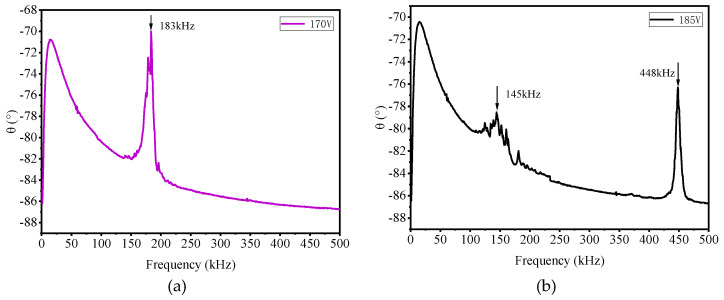
Pull-in voltage test results: (**a**) DC voltage 170 V, and (**b**) DC voltage 185 V.

Figure 6 shows the frequency–phase relationship under different DC voltages. When the DC voltage is 170 V, the resonant frequency is 183 kHz. When the DC voltage reaches 185 V, the amplitude of the resonance peak at 448 kHz increases significantly, while the resonance peak near 150 kHz almost completely disappears. This indicates that most of the cells on the CMUT undergo pull-in effects. The pull-in voltage is between 170 V and 185 V.

#### 2.3.4. Transmitting Characteristics Testing

**Figure 7:**
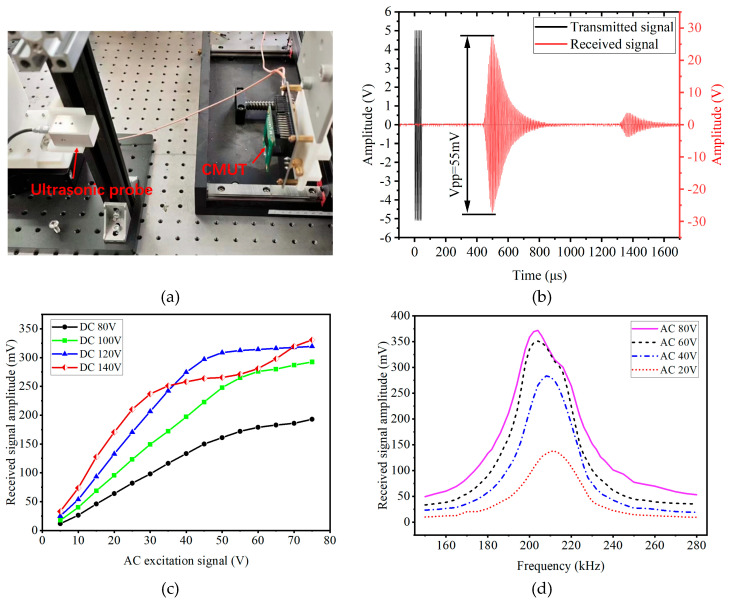
CMUT emission characteristics test results: (**a**) Performance testing setup. (**b**) Excitation and reception signals at DC 120 V. (**c**) Impact of AC excitation on transmitting performance. (**d**) Impact of AC excitation on resonance characteristics.

The transmitting performance of the CMUT array was characterized using a 200 kHz air-coupled ultrasonic probe (Japan Probe AR0.2K14 × 20). The CMUT device was fixed on a motorized translation stage controlled by a computer, while the air-coupled probe was mounted on a platform with the two transducers placed 15 cm apart, as shown in Figure 7a.

Figure 7b illustrates the excitation and reception signal of the CMUT. DC biases of 80 V, 100 V, 120 V, and 140 V were applied to the CMUT array, corresponding to AC excitation frequencies of 220 kHz, 216 kHz, 212 kHz, and 204 kHz. A series of AC signals from 5 V to 75 V, in 5 V increments, with 10 cycles, was applied to the device. The test results are shown in Figure 7c. When the AC amplitude is small, the emission intensity of the device increases linearly; however, the growth rate slows at higher amplitudes due to membrane–substrate contact, consistent with simulation.

Figure 7d shows the impact of different AC voltage amplitudes on the CMUT emission bandwidth under a DC bias of 120 V. As the AC voltage increases, the frequency point corresponding to the maximum emission intensity of the device decreases. Under AC voltages of 20 V, 40 V, 60 V, and 80 V, the frequencies corresponding to the maximum emission intensity are 212 kHz, 208 kHz, 204 kHz, and 204 kHz, respectively. These test results align with the trends observed in simulations.

#### 2.3.5. Transmission Distance Testing

To measure the relationship between transmission distance and reception signal strength for two CMUTs, the emitting CMUT was fixed on a platform while the receiving CMUT was mounted on the motorized translation stage, moving along the axis of the transducer. The two CMUTs were excited with five sinusoidal pulses with 20 V at 206 kHz, at a DC voltage of 130 V.

**Figure 8:**
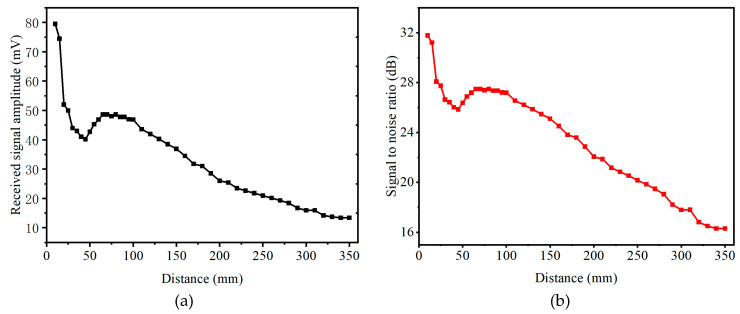
Relationship between received signal and distance: (**a**) relationship between signal amplitude and distance, and (**b**) relationship between reception SNR and distance.

Figure 8a shows the relationship between the amplitude of the received signal and distance. The received signal amplitude first decreased and then increased from 10 mm to 65 mm due to near-field effects. The noise of the received signal was 2.05 mV, leading to the calculation of the signal-to-noise ratio (SNR), as shown in Figure 8b. At a distance of 350 mm, the SNR was still 16.3 dB, with 13.4 mV. The experimental results demonstrate that the CMUT array has excellent transmission performance.

## 3. CMUT Temperature Measurement Method

Acoustic temperature measurement utilizes the functional correspondence between the temperature and sound velocity. By measuring the transmission time of sound waves, the speed of sound along the transmission path is calculated, and then the temperature of the medium is inversely calculated. The relationship between sound velocity and medium gas temperature is shown in Formula (1).

(1) $$v=\sqrt{\frac{\gamma RT}{m}}=Z\sqrt{T}$$

Among them, *v* represents the speed of sound, *γ* represents the adiabatic exponent, *R* is the ideal gas constant, *m* represents the molar mass, *T* represents the thermodynamic temperature, and the unit is *K*.

Assuming the distance between two transducers is *d*, the transmission time of sound waves is *t*, and the average temperature *T_a_* on this sound path:

(2) $$T_{a}={\left(\frac{d}{Zt}\right)}^{2}$$

However, this formula can only calculate the average temperature along the sound path. In order to measure the temperature distribution in non-uniform areas, it is necessary to combine temperature reconstruction algorithms and multi-channel acoustic path data to complete the reconstruction of the temperature field.

**Figure 9:**
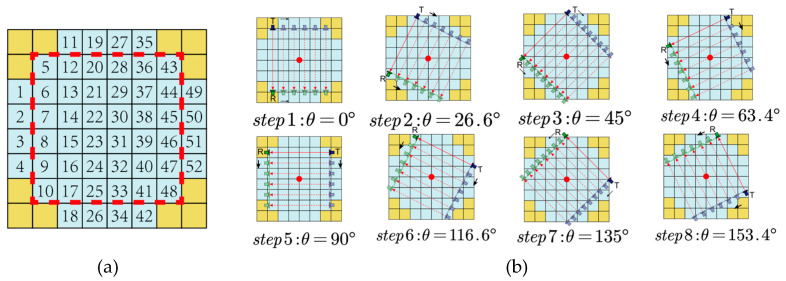
Schematic diagram of temperature field reconstruction scanning scheme: (**a**) grid division of measurement area and (**b**) mechanical scanning scheme.

The specific steps of the temperature field reconstruction method designed in this paper are as follows:

- Step 1: Grid division. The measurement area is divided into grids with uniform temperature distribution within each grid based on the measurement scenario and resolution. The more grids there are, the higher the resolution, but the required number of sound paths will also increase, corresponding to a larger computational load. Therefore, it is necessary to select the appropriate number of grids according to the imaging requirements. When setting sound paths, it is necessary to ensure that all sound paths cover the entire grid area as much as possible. And it is necessary to ensure that the sound path does not overlap with the boundary of the grid. Taking into account factors such as complexity of the reconstruction algorithm and experimental feasibility, the measured areas were divided into a 6 × 6 grid as shown in Figure 9a. Among them, the yellow grid is the non-measurement grid, the blue grid containing numbers is the measurement grid, and the grid with red dashed boxes is the reconstruction area.
- Step 2: Scanning scheme. This article uses two sets of CMUT transducers to obtain acoustic projection data from different orientations through a scanning motion that combines mechanical translation and rotation. Figure 9b shows the scanning scheme. Linear scanning was performed on 8 different angle directions, resulting in a total of 58 paths. There were 52 paths in the blue grid area, and the number of sound paths was greater than the number of grids. The sound wave reception signals on the sound path at different positions were obtained by receiving the CMUT chip.
- Step 3: Temperature field reconstruction. Obtain the received signal flight time of all sound paths through cross-correlation and other time delay estimation algorithms. Due to the uniform temperature distribution of each grid, the flight time of a single path can be rewritten as Formula (3).
  (3) $$tof_{k}=\sum_{i=1}^{q}a_{i}\Delta S_{ki}$$

Among them, *a_i_* represents the reciprocal of the sound velocity in the *i_th_* grid, and *∆S_ki_* represents the distance that the *k_th_* sound path passes through the *i_th_* grid. Where *i* = 1,2... *q*, *q* is the total number of grids. And *k* = 1,2... *m*, where *m* is the total number of sound paths. Convert different data into matrix form:

(4) $$A={\left[a_{1}\;a_{2}\ldots \;a_{q}\right]}^{T}$$

(5) $$P={\left[tof_{1}\;tof_{2}\ldots \;tof_{m}\right]}^{T}$$

(6) $$S=\left[\begin{array}{cccc} \Delta S_{11} & \Delta S_{12} & \ldots & \Delta S_{1q}\\ \Delta S_{21} & \Delta S_{22} & \ldots & \Delta S_{2q}\\ \vdots & \vdots & & \vdots \\ \Delta S_{m1} & \Delta S_{m1} & \ldots & \Delta S_{mq} \end{array}\right]$$

Among them, *A* is the column vector of the reciprocal of the sound velocity corresponding to the grid temperature. *P* is the column vector of flight time data for each sound path collected from the temperature field, and *S* is the reconstruction matrix. When solving the temperature field distribution, the time matrix *P* of the sound path can be obtained through measurement, and the reconstruction matrix S is determined by grid division and the sound path.

(7) $$A=S^{-1}P$$

The temperature distribution expression of the two-dimensional temperature measurement plane area based on the correspondence between temperature and sound velocity is derived, as shown in Formula (8):

(8) $$T(x,y)=\frac{1}{A^{2}Z^{2}}$$

## 4. Experiment Validation

### 4.1. Uniform Temperature Field

**Figure 10:**
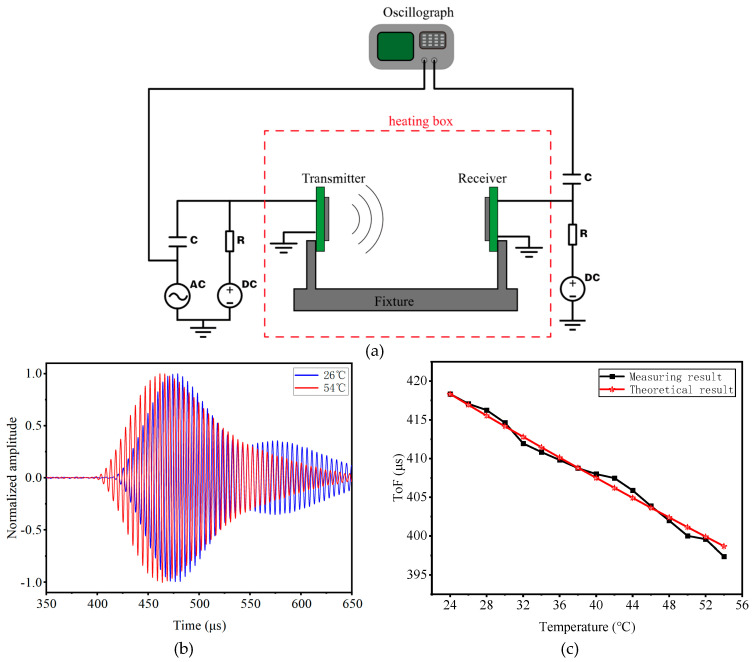
Measurement results of uniform temperature field: (**a**) Schematic diagram of experimental setup. (**b**) Received signal waveform. (**c**) Arrival time data at different temperatures.

Firstly, the feasibility of the device and detection principle is verified through a uniform temperature field. Fix CMUT onto the fixture and place it in the heating box, as shown in Figure 10a. The distance between two CMUT chips is 144.52 mm, the DC bias voltage is 140 V, the excitation signal is 204 kHz, the number of pulse trains is 10, and the peak-to-peak value is 20 V. Control the temperature of the heating box and record the waveform of the received signal under different temperatures. Figure 10b shows the sound waves under different temperature conditions. The received signal at 54 °C is significantly ahead of that at 26 °C, indicating that the sound velocity at 54 °C is faster than that at 26 °C. Figure 10c shows the arrival time of sound waves at different temperatures, and it can be found that the measurement results are consistent with the theoretical results.

### 4.2. Scanning of Temperature Field Cross-Section

**Figure 11:**
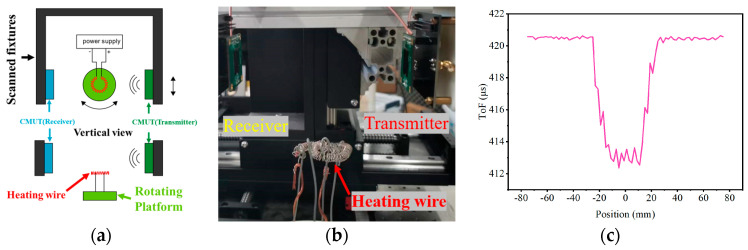
Temperature field cross-section measurement experiment: (**a**) Device schematic diagram. (**b**) Physical device diagram. (**c**) Measurement results.

In order to further investigate the detection capability of the method proposed based on CMUT for the non-uniform temperature field, the sound velocity changes at different positions were measured along the cross-section above the heating wire. The experimental setup is shown in Figure 11a,b. Using the middle position of the heating wire as the scanning center point, a 2 mm step size and a total scanning length of 150 mm are used to scan the temperature measurement plane in a straight line. Figure 11c shows the measurement results. When the scanning position is in the range of −25 mm to 25 mm, the transmission time of sound waves is significantly reduced, corresponding to the high-temperature convection area directly above the heating wire. The transmission time of other scanning areas fluctuates within the range of 420.62 μs, corresponding to the low-temperature area.

### 4.3. Temperature Field Reconstruction

**Figure 12:**
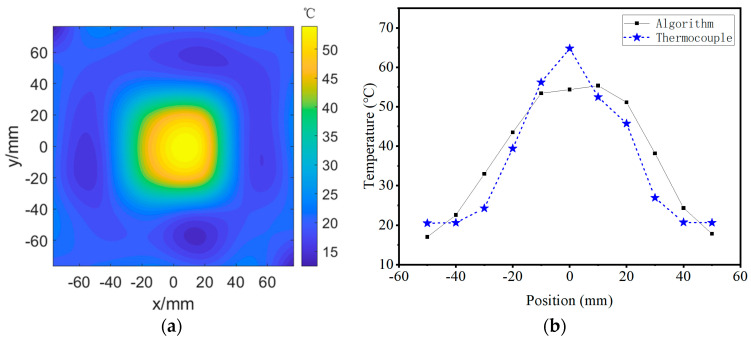
Experimental results of temperature field reconstruction. (**a**) temperature field imaging and (**b**) result comparison.

Based on the scanning scheme proposed in Section 3, the horizontal plane 5 cm above the electric heating wire was scanned and measured. The temperature measurement area was divided into a 6 × 6 grid, with each individual grid cell being 25.55 mm × 25.55 mm. The total temperature measurement area was 153.28 mm × 153.28 mm, and the reconstruction result of the temperature field is shown in Figure 12a. The imaging results show that the temperature in the middle area is the highest, around 50 °C. In order to compare the relationship between the reconstructed temperature field results and the actual temperature, the thermocouple measurement values were compared with the reconstructed results, as shown in Figure 12b. The measurement results have a consistent trend with the reconstruction results, which can reflect the temperature information within the measured range. However, there is still a certain degree of error between the reconstruction results and the thermocouple measurement values, which may be caused by the insufficiently fine grid division of the imaging area, resulting in unsatisfactory resolution and accuracy of the final imaging results for real temperature field detection. In addition, during the measurement process, there is air flow above the heating wire, which can cause some disturbance to the measurement of flight time and increase the reconstruction error of the temperature field.

## 5. Conclusions

This article focuses on the demand for non-contact rapid temperature measurement and studies the air-coupled CMUT devices for acoustic temperature measurement technology. The device design and fabrication were completed, and the working frequency of the transducer was 220 kHz. The diameter of the CMUT cell was designed to be 400 μm, the membrane thickness was 10 μ m, and the cavity depth was 5 μm. The device characteristics were tested and characterized, proving that the air-coupled CMUT device has good transmission and reception performance. A temperature field reconstruction method using mechanical scanning was proposed, and imaging of the temperature field above the electric heating wire was completed using a temperature measurement experimental device, validated by thermocouple measurement. The causes of errors and outliers were analyzed. 

This study indicates that the air-coupled CMUT device has advantages for ultrasonic temperature field imaging. Due to its small size, light weight, high frequency, ability to adapt to harsh environments such as high temperature, and better matching with air acoustic impedance, air-coupled CMUT has significant promise in applications that require convenience, small spatial size, and high measurement accuracy. Future research will improve the detection sensitivity of CMUT by improving the packaging and electromagnetic shielding. Additionally, measurement accuracy will be further improved by reducing scanning grid size, enhancing the accuracy of TOF algorithms, and optimizing temperature field reconstruction algorithms.

## Figures and Tables

**Table 1:** CMUT structural parameters.

Parameter	Value/um
Membrane radius, *rm*	400
Membrane thickness, *hm*	10
Insulating layer thickness, *hi*	2.2
Cavity depth, *hg*	5
Metal electrode diameter, *re*	200
Metal electrode thickness, *he*	0.2

## Data Availability

The original contributions presented in the study are included in the article; further inquiries can be directed to the corresponding author.
